# Supplementary material for: Identification of PCB congeners and their thresholds associated with diabetes using decision tree analysis
Source: Sci Rep. 2023 Oct 26;13:18322. doi: 10.1038/s41598-023-45301-1 (PMC10603165; doi:10.1038/s41598-023-45301-1)
Supplement: Supplementary file 1 — Supplementary Information. [file 41598_2023_45301_MOESM1_ESM.docx]

**Supplement**

**Identification of** **PCB Congeners and their Thresholds associated with Diabetes using Decision Tree Analysis**

Tuo Lan^1^, Buyun Liu^2^, Wei Bao^2^, Peter S. Thorne^1,3*^

^1^Department of Occupational and Environmental Health, College of Public Health, University of Iowa, Iowa City, Iowa, USA

^2^Division of Life Sciences and Medicine, University of Science and Technology of China, China

^3^Human Toxicology Program, University of Iowa, Iowa City, Iowa, USA

**Correspondence Author***

Peter S. Thorne, MS, PhD

University of Iowa College of Public Health

100 CPHB, 145 N. Riverside Dr.

Iowa City, IA 52242 USA

Email: [peter -thorne@uiowa.edu](mailto:peter-thorne@uiowa.edu)

Peter S. Thorne: ORCID: 0000-0002-5045-0929

Supplemental Codes for Decision Tree Analysis

# Split the data into training and test set
set.seed(123)
training.samples <- createDataPartition(PCB$DIABETES,p = 0.7, list = FALSE)
train.data <- PCB[training.samples, ]
test.data <- PCB[-training.samples, ]

# Build the model
model1 <- rpart(DIABETES ~ PCB28+PCB44+PCB49+PCB52+PCB66+PCB74+PCB81
 +PCB87+PCB99+PCB101+PCB105+PCB110+PCB118+PCB126
 +PCB128+PCB138and158+PCB146+PCB149+PCB151+PCB153+PCB156+PCB157+PCB167+PCB169
 +PCB170+PCB172+PCB177+PCB178+PCB180+PCB183
 +PCB187+PCB189+PCB194+PCB195+PCB196and203+PCB199
 +PCB206+PCB209,
 control=(xval=10),
 data = train.data,
 metho="class")

# Make predictions on the test data confusion matrix
predicted.classes <- predict(model1, test.data, type = "class")
table_mat <- table(test.data$DMCASE, predicted.classes )
table_mat

## predicted.classes
## No yes
## 0 286 32
## 1 42 6

# Compute model accuracy rate on test data
#unprune model
accuracy_Test <- sum(diag(table_mat)) / sum(table_mat)
print(paste('Accuracy for test', accuracy_Test))

## [1] "Accuracy for test 0.797814207650273"

#cost-complexity pruning
printcp(model1)

##
## Classification tree:
## rpart(formula = DIABETES ~ PCB28 + PCB44 + PCB49 + PCB52 + PCB66 +
## PCB74 + PCB81 + PCB87 + PCB99 + PCB101 + PCB105 + PCB110 +
## PCB118 + PCB126 + PCB128 + PCB138and158 + PCB146 + PCB149 +
## PCB151 + PCB153 + PCB156 + PCB157 + PCB167 + PCB169 + PCB170 +
## PCB172 + PCB177 + PCB178 + PCB180 + PCB183 + PCB187 + PCB189 +
## PCB194 + PCB195 + PCB196and203 + PCB199 + PCB206 + PCB209,
## data = train.data, method = "class", control = (xval = 10))
##
## Variables actually used in tree construction:
## [1] PCB101 PCB105 PCB126 PCB128 PCB138and158
## [6] PCB153 PCB177 PCB206 PCB44 PCB74
## [11] PCB99
##
## Root node error: 113/858 = 0.1317
##
## n= 858
##
## CP nsplit rel error xerror xstd
## 1 0.013274 0 1.00000 1.0000 0.087659
## 2 0.011799 12 0.77876 1.2124 0.094952
## 3 0.010000 15 0.74336 1.2124 0.094952

plotcp(model1)


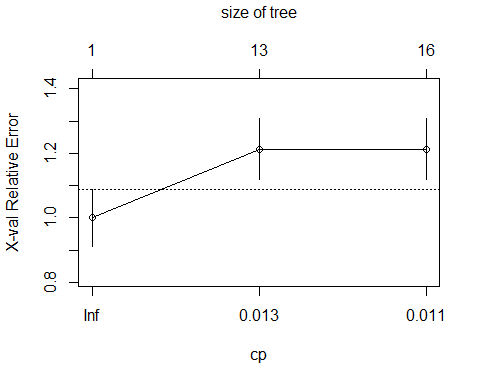


#prune tree and select control2
control2 <- rpart.control(minsplit = 30,
 minbucket = 15,
 xval=10,
 maxdepth = 6,
 cp = 0.013274)
model2 <- rpart(DIABETES ~ PCB28+PCB44+PCB49+PCB52+PCB66+PCB74+PCB81
 +PCB87+PCB99+PCB101+PCB105+PCB110+PCB118+PCB126
 +PCB128+PCB138and158+PCB146+PCB149+PCB151+PCB153+PCB156+PCB157+PCB167+PCB169
 +PCB170+PCB172+PCB177+PCB178+PCB180+PCB183
 +PCB187+PCB189+PCB194+PCB195+PCB196and203+PCB199
 +PCB206+PCB209,
 data = train.data,
 metho="class",control = control2)
print(model2)

## n= 858
##
## node), split, n, loss, yval, (yprob)
## * denotes terminal node
##
## 1) root 858 113 No (0.86829837 0.13170163)
## 2) PCB126< 0.02495 555 39 No (0.92972973 0.07027027) *
## 3) PCB126>=0.02495 303 74 No (0.75577558 0.24422442)
## 6) PCB101>=0.72 259 55 No (0.78764479 0.21235521)
## 12) PCB49< 1.395 116 16 No (0.86206897 0.13793103) *
## 13) PCB49>=1.395 143 39 No (0.72727273 0.27272727)
## 26) PCB151>=0.465 85 16 No (0.81176471 0.18823529) *
## 27) PCB151< 0.465 58 23 No (0.60344828 0.39655172)
## 54) PCB149< 0.735 27 6 No (0.77777778 0.22222222) *
## 55) PCB149>=0.735 31 14 yes (0.45161290 0.54838710)
## 110) PCB169< 0.02065 15 5 No (0.66666667 0.33333333) *
## 111) PCB169>=0.02065 16 4 yes (0.25000000 0.75000000) *
## 7) PCB101< 0.72 44 19 No (0.56818182 0.43181818)
## 14) PCB49< 0.645 29 9 No (0.68965517 0.31034483) *
## 15) PCB49>=0.645 15 5 yes (0.33333333 0.66666667) *

# Make predictions on the test data
predicted.classes <- predict(model2, test.data, type = "class")
table_mat <- table(test.data$DMCASE, predicted.classes )
table_mat

## predicted.classes
## No yes
## 0 303 15
## 1 43 5

# Compute model accuracy rate on test data
accuracy_Test <- sum(diag(table_mat)) / sum(table_mat)
print(paste('Accuracy for test', accuracy_Test))

## [1] "Accuracy for test 0.841530054644809"
