# Supplementary material for: Identification of PCB congeners and their thresholds associated with diabetes using decision tree analysis
Source: Sci Rep. 2023 Oct 26;13:18322. doi: 10.1038/s41598-023-45301-1 (PMC10603165; doi:10.1038/s41598-023-45301-1)
Supplement: Supplementary file 2 — Supplementary Table 1. [file 41598_2023_45301_MOESM2_ESM.docx]

Supplemental table 1. Sensitivity analysis excluding diabetes type I. Multivariable-adjusted odd ratios (ORs) and 95% confidence intervals (CIs) of diabetes by the combined associations of PCB congeners, NHANES 2003–2004.

| PCB profiles | No. of exposure/ No. of subgroup population | Reference | ORs (95% CI) |
| --- | --- | --- | --- |
| PCB 126 > 0.025 | 396/1188 | PCB 126 ≤ 0.025 |  |
| Basic model1 |  | 1 | 2.77 (1.49-5.13) |
| Fully adjusted model2 |  | 1 | 2.94 (1.61-5.34) |
| PCB 126 > 0.025  & PCB 101 < 0.72 | 62/396 | PCB 126 > 0.025  & PCB 101 ≥ 0.72 |  |
| Basic model1 |  | 1 | 2.72 (1.02-7.29) |
| Fully adjusted model2 |  | 1 | 3.74 (1.36-10.3) |
| PCB 126 >0.025  & PCB 101 <0.72  & PCB 49 ≥0.65 | 25/62 | PCB 126 > 0.025  & PCB 101 < 0.72  & PCB 49 < 0.65 |  |
| Basic model1 |  | 1 | 4.99 (1.81-13.8) |
| Fully adjusted model2 |  | 1 | 1.25 (0.16-9.73) |
| PCB 126 >0.025  & PCB 101 ≥ 0.72  & PCB 49 ≥ 1.4 | 181/334 | PCB 126 > 0.025  & PCB 101 ≥ 0.72  & PCB 49 < 1.4 |  |
| Basic model1 |  | 1 | 1.18 (0.45-3.09) |
| Fully adjusted model2 |  | 1 | 2.07 (0.68-6.32) |
| PCB 126 >0.025  & PCB 101 ≥ 0.72  & PCB 49 ≥ 1.4  & PCB 151 <0.47 | 72/181 | PCB 126 > 0.025  & PCB 101 ≥ 0.72  & PCB 49 ≥ 1.4  & PCB 151≥ 0.47 |  |
| Basic model1 |  | 1 | 3.02 (0.98-9.26) |
| Fully adjusted model2 |  | 1 | 2.35 (0.79-6.95) |
| PCB 126 > 0.025  & PCB 101 ≥ 0.72  & PCB 49 ≥ 1.4  & PCB 151 <0.47  & PCB 49 ≥ 0.74 | 36/72 | PCB 126 > 0.025  & PCB 101 ≥ 0.72  & PCB 49 ≥ 1.4  & PCB 151 <0.47  & PCB 49 < 0.74 |  |
| Basic model1 |  | 1 | 6.44 (1.26-32.9) |
| Fully adjusted model2 |  | 1 | Very large or infinite3 |
| PCB 126 > 0.025  & PCB 101 ≥ 0.72  & PCB 49 ≥ 1.4  & PCB 151 <0.47  & PCB 49 ≥ 0.74  & PCB 169 ≥ 0.021 | 18/36 | PCB 126 > 0.025  & PCB 101 ≥ 0.72  & PCB 49 ≥ 1.4  & PCB 151 <0.47  & PCB 49 ≥ 0.74  & PCB 169 < 0.021 |  |
| Basic model1 |  | 1 | 11.5 (1.67-78.9) |
| Fully adjusted model2 |  | 1 | Very large or infinite3 |

^1^Basic model was adjusted for age, sex, race/ethnicity

^2^ Full model was adjusted for age, sex, race/ethnicity, BMI, education level, Family income to poverty ratio, smoking status, alcohol intake, physical activity level, 2010 healthy eating index, and family history of diabetes.

^3^ The fully adjusted odd ratio was very large due to the small sample size. Some covariates had few observations in the sub-category group.
